# Unique multiple paternity in the endangered big‐headed turtle (*Platysternon megacephalum*) in an ex situ population in South China

**DOI:** 10.1002/ece3.5528

**Published:** 2019-08-15

**Authors:** Shiping Gong, Liushuai Hua, Yan Ge, Dainan Cao

**Affiliations:** ^1^ Guangdong Key Laboratory of Animal Conservation and Resource Utilization Guangdong Public Laboratory of Wild Animal Conservation and Utilization Guangdong Institute of Applied Biological Resources Guangzhou China

**Keywords:** captive breeding, male hierarchy, microsatellite marker, mimic natural habitat, multiple paternity, Platysternon megacephalum

## Abstract

Understanding the mating system and reproductive strategies of an endangered species is critical to the success of captive breeding. The big‐headed turtle (*Platysternon megacephalum*) is one of the most threatened turtle species in the world. Captive breeding and reintroduction are necessary to re‐establish wild populations of *P. megacephalum* in some of its historical ranges in China, where the original populations have been extirpated. However, the captive breeding of *P. megacephalum* is very difficult and this may be due to its mysterious reproductive strategies and special behavior (e.g., aggressive temperament and territoriality). In this study, we achieved successful captive breeding of *P. megacephalum* by creating a habitat that mimics natural conditions and then investigated its mating system using microsatellite makers. A total of 16 clutches containing 79 eggs of *P. megacephalum* were collected, and 52 were hatched successfully over two breeding seasons. Of the 15 effective clutches, 6 clutches (40%) exhibited multiple paternity. There was no significant correlation between clutch size and multiple paternity, and no significant difference in hatching success between multiple‐sired and single‐sired clutches. However, there was significant correlation between male body size and the number of offspring, with higher‐ranked males contributing to more clutches. Our results provide the first evidence of multiple paternity and male hierarchy in *P. megacephalum*. These findings suggest that multiple paternity and male hierarchy should be considered in captive breeding programs for *P. megacephalum*, and creating a habitat that mimics natural conditions is an effctive way to achieve successful captive breeding and investigate the mating systems of this species.

## 1 INTRODUCTION

Turtles are currently one of the most threatened vertebrate taxa. About 59% of 356 extant turtle species are threatened, and 114 (32.0%) are classified as critically eandanged or endangered (Rhodin et al., 2017). Asian turtles, especially Chinese turtles, are the most seriously threatened turtle taxa worldwide (Altherr & Freyer, 2000; Gong et al., 2017). The big‐headed turtle (*Platysternon megacephalum*), as the sole member of the family Platysternidae, is an endangered species and one of the most threatened turtle species in the world (Rhodin et al., 2017). Because of over‐harvesting and habitat destruction, only a few wild populations of *P. megacephalum* remain (Gong et al., 2017; Sung, Karraker, & Hau, 2013). Captive breeding and reintroduction are important approaches to improve the recovery of *P. megacephalum* in some of its historical ranges (China, Vietnam, Thailand, Myanmar, Laos, and Cambodia, Rhodin et al., 2017), where the original populations have been extirpated but the natural habitat still remains (Gong et al., 2017; Shen, Pike, & Du, 2010).

In recent decade years, big‐headed turtles are often found in illegal markets (Gong, Chow, Fong, & Shi, 2009; Gong et al., 2017). When these individuals are confiscated, they provide a source of individuals for ex situ conservation. Successful reproduction in ex situ population is a necessary first step in the recovery of the big‐headed turtles. Regretfully, however, the captive breeding of *P. megacephalum* is difficult and has not been successful in recent decades (Shelmidine, Murphy, & Massarone, 2016; Wei, Gong, Shi, & Li, 2016). Under captive conditions, big‐headed turtles usually fight and cause severe disability or even death. This may be due to its specific reproductive strategies, or behavioral characteristics, such as aggressive temperament and territoriality (Gong et al., 2013). Currently, little is known about the mating systems and reproductive strategies of *P. megacephalum*, meaning there is no scientific basis for captive breeding.

Investigating the mating systems of *P. megacephalum* could improve our understanding of the species' reproductive ecology and aid captive breeding programs. However, it is almost impossible to investigate the mating systems of *P. megacephalum* in the wild because this species lives in rocky mountain streams (Shen et al., 2010; Sung, Hau, & Karraker, 2015), which makes behavioral observation, egg collection, and study of mating systems extremely difficult. Even if the mating behavior can be observed, it is difficult to determine the genetic mating system because of potential extra‐pair copulation, sperm competition, and unsuccessful mating (Pearse & Avise, 2001; Rossi Lafferriere et al., 2016). In the past 30 years, molecular markers have provided an effective means to infer animal mating systems (Pearse & Avise, 2001; Rossi Lafferriere et al., 2016) and microsatellite DNA makers are a powerful genetic tool for the analysis of parentage (Jones & Ardren, 2003; Roques, Díaz‐Paniagua, & Andreu, 2004).

In this study, we achieved successful captive breeding of *P. megacephalum* by creating a habitat that mimics natural conditions (Gong et al., 2012), which gave us an opportunity to investigate its mating systems using microsatellite DNA makers. The main goals of this study were to reveal the characteristics of mating systems and provide a scientific basis for captive breeding of *P. megacephalum*.

## 2 MATERIALS AND METHODS

### 2.1 Experimental animals and imitating nature habitat

Ethics approval was granted by the the Animal Care & Welfare Committee, Guangdong Institute of Applied Biological Resources (permit numbers: No. GIABR20100106). A total of 20 adult big‐headed turtles (8 males, 12 females) from South China were used in this study. Detailed information for each animal is provided in Table 1. The turtles were seized from illegal animal markets and then raised in a turtle conservation center in Huizhou, Guangdong, South China. To encourage breeding, an mimicing natural habitat (resembling natural ponds) was created near a natural mountain stream. Each pond was rectangular, with an area of 20 square meters (4 m × 5 m). Half of the area was a water pool, and the other half was land. Flowing mountain spring water was introduced into the pool via pipes. The water temperature was 12–26°C during the year, the pH was approximately 6.4, and the water depth was 15–30 cm. Some stone caves were established in the pools as shelters for the turtles. The plants (e.g., *Acorus tatarinowii*, *Blechnum orientale*) from the natural habitats of the turtles were transplanted into the land section of the pools to create suitable nest microenvironments. Some living stream shrimps and small fish were introduced to the ponds to provide food for the turtles. In addition, fresh loaches, fish, river snails, and earthworms were also offered as supplementary food 2–3 times each week. The 20 turtles were divided into two groups and put into two ponds (pond I, pond II) separately (Table 1). In pond I, three males exhibiting significant differences in weight and body size were grouped with seven females, to help us to understand the effects of body size on social ranking. In pond II, five males and five females with only minor body size variations were grouped together.

**Table 1:** Body measurements of the 20 *Platysternon megacephalum* used in this study

Pond #	Individual ID	Sex	Age (in 2012)	Body weight (g)	Carapace length (mm)	Plastron length (mm)
Pond I	F1‐1	Male	Over 10	1,511	209	148
	F1‐2	Male	Over 10	1,074	192	139
	F1‐3	Male	7	609	158	123
	M1‐1	Female	Over 10	538	145	113
	M1‐2	Female	Over 10	396	134	109
	M1‐3	Female	Over 10	397	132	102
	M1‐4	Female	Over 10	331	125	103
	M1‐5	Female	Over 10	345	122	102
	M1‐6	Female	Over 10	373	123	106
	M1‐7	Female	Over 10	326	124	95
Pond II	F2‐1	Male	6	470	145	107
	F2‐2	Male	6	412	141	111
	F2‐3	Male	6	426	138	107
	F2‐4	Male	6	428	138	104
	F2‐5	Male	6	368	137	105
	M2‐1	Female	6	364	130	101
	M2‐2	Female	6	341	126	104
	M2‐3	Female	6	315	120	100
	M2‐4	Female	6	251	117	93
	M2‐5	Female	6	239	114	91

Note

Age was roughly estimated on the basis of the rings on the scute.

Abbreviations: F, father candidate; M, mother candidate.

### 2.2 Sampling

From our observations, the laying season for *P. megacephalum* is from June to August, so we searched for eggs every day during this time. To prevent potential damage by snakes and mice, a cylindrical wire mesh cover was immediately placed over the nest. A total of 16 clutches of *P. megacephalum* were found in pond I (13 clutches) and pond II (3 clutches) during 2012–2013. Automatic temperature recorders (Taiwan Hengxin: AZ8829) were used to record the nest temperatures during incubation. After hatching, the newborn turtles were transported to housing for care. Tissue samples of the offspring were taken from the tail tips (0.5 cm). Oral swab samples, rather than tissue samples, were collected from the 20 parental turtles to reduce the potential sampling damage as much as possible. Tissue samples and oral swab samples were stored at −20°C in 95% ethanol.

### 2.3 Microsatellite genotyping

Genomic DNA was extracted from tail tissue and swab samples using the phenol–chloroform protocol and salting‐out method, respectively (Sambrook & Russell, 2001). All individuals were genotyped at nine, previously developed microsatellite loci for *P. megacephalum* (Table 2). The PCRs were performed in volumes of 15 μl, which contained 1× Buffer, 1.5 mmol/L MgCl_2_, 200 μmol/L dNTPs, 0.2 μmol/L Primer (each), and 0.02 U/μl Taq DNA polymerase. The PCR conditions were 94°C for 3 min for denaturation; 34 cycles of 30 s denaturation at 94°C; 55°C for 30 s for annealing; 72°C for 30 s for extension; and final extension for 5 min at 72°C. The PCR products were separated with 10% polyacrylamide gel electrophoresis (PAGE) and were visualized using silver staining (Zhang, Wang, Chen, Lan, & Lei, 2007).

**Table 2:** The nine microsatellite markers used in this study

Loci	Primers sequences (5′‐3′)
Pme14	F: CTGTGCACAGCAGACATG; R: AGCTACTGCCTAGGTCCT
Pme42	F: GTACCAGGCTGTAGGGG; R: GTTGGGGTTGTAGTTCTCA
Pme56	F: GATGCTAAACGCTCCTAAA; R: CATATGGTCCTCTGTGGG
Pme59	F: GATACGCACTCGCACTCA; R: AAGGCAATTACTTTTCTCCTC
Pme61	F: AGAAAGGACCCATCAAACA; R: GGGACTCACCCTCAACTAA
Pme112	F: TTACAGGGCTCGCTTTC; R: GTGTCTGCTGGTGACGG
Pme128	F: TGGGAGGAGACGGGGCATG; R: TGGGGTGGGCAGAAGGGTG
Pme156	F: CTTGGCAGTCTGGCTTCA; R: TTCCCATCCACCCCTTT
Pme165	F: TGCGGTGTTATGAAAGAG; R: TTATGTTCCAAGTTGTCCC

Note

The nine microsatellite markers for *Platysternon megacephalum* developed by Hua et al. (2014).

### 2.4 Paternity analysis

The average allele numbers, mean expected heterozygosity, and polymorphic information content (PIC) of the microsatellite loci were calculated using CERVUS version 3.0 (Kalinowski, Taper, & Marshall, 2007). The parentage analysis module available in CERVUS was used to calculate the success of assignment of candidate parents to the offspring. CERVUS uses a log (base e) likelihood algorithm to calculate the likelihood ratio (LOD) of a candidate male being the true parent compared with an random male. LOD scores are calculated for all possible sires, and the difference between the two most likely candidates (delta) is calculated and provides an indication of the reliability of the assignment. The delta score is calculated at a confidence level of 95% and corresponds to an estimated frequency of false positives of 5% (Moon, McCoy, Mushinsky, & Karl, 2006; Roques et al., 2004). First, the candidate mothers of each clutch were verified. Once the candidate mother and the offspring were matched, the paternal alleles were deduced from the comparison of both maternal and offspring genotypes. We assumed that clutches with more than two paternal alleles were multiple paternity. SPSS 17.0 Statistical Analysis Software and Pearson correlation coefficients were used to analyze correlations between variables (e.g., body size of female or male, clutch size, and the number of offspring).

### 2.5 Social behavioral observations

To explore the influence of social hierarchies on the mating system, the social behaviors of big‐headed turtles were observed every 2–3 days from April to November during 2012–2013. All turtles were marked for individual recognition with a white or red number code painted on the carapace. In order to reflect an individual's social rank, the territory size and the eating order of each turtle were observed. Based on observation, females in pond I seem to have no territory (or territorial behavior) and they can move freely anywhere in the pool. The males in pond I have their territories, and territorial behavior occurs only among males. The males in pond II seem to have no territory (or territorial behavior), and they can move freely anywhere in the pool. Except for the three males in pond I, the feeding order of other turtles in pond I and pond II seems random. So, in this study, we only analyzed the territory and eating order in males in pond I. The territory of each male in pond I was approximate estimated by the irregular polygons generated by all occurrence site connections at the edge of active range. The feeding order of the males in pond I was analyzed by chi‐square test based on the behavioral observation data.

## 3 RESULTS

### 3.1 Egg hatching

**Figure 1:**
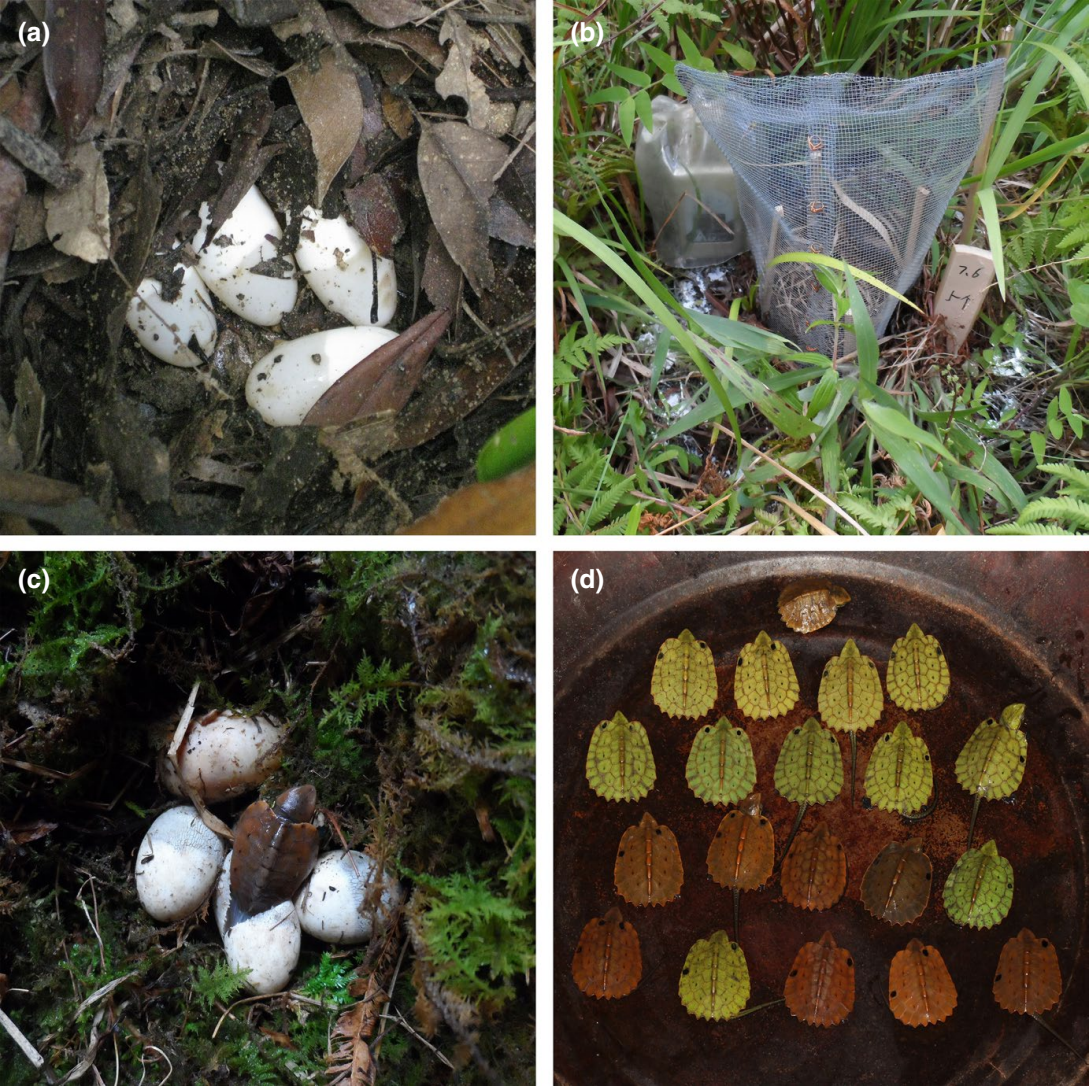
Eggs, clutches, and newborn hatchlings of *Platysternon megacephalum*. (a) A clutch with four eggs was found on 3 July 2013. (b) Wire mesh was used to protect the clutch from potential damage by snakes and mice. (c) One of the newborn hatchlings emerging from its shell. (d) The newborn hatchlings were collected in a basin, and each of the hatchlings was given an ID mark on the carapce

A total of 16 clutches with 79 eggs (mean 5 eggs per nest) were collected, of which 13 clutches (63 eggs) were collected from pond I and 3 clutches (16 eggs) from pond II. The fluctuation of nest temperatures during the incubation period ranged from 11.5 to 32.5°C (mean 22.4°C), and the incubation period ranged from 78 to 97 days. The fluctuation of relative humidity in the nest during the incubation period ranged from 63.2% to 100% (mean 97%). Nine of the eggs were destroyed by ants during the incubation period, and 18 eggs failed to develop. A total of 52 offspring successfully hatched from 15 clutches, and the total hatching rate was 74.3% (Table 3). Some of the nests, eggs, and hatchlings are shown in Figure 1.

**Table 3:** Egg collection, hatching, and paternity assignment of 16 clutches of *Platysternon megacephalum*

Year	Clutch ID	Pond #	*N*_1_/*N*_2_/*N*_3_/*N*_4_	Hatching success (%)	Inferred mother ID	Inferred father ID	Number of offspring
2012	2012‐1‐1^a^	I	4/2/0/2	100	M1‐6	F1‐1 F1‐2	1 1
	2012‐1‐2	I	5/0/0/5	100	M1‐3	F1‐1	5
	2012‐1‐3	I	8/4/0/4	100	M1‐2	F1‐2	4
	2012‐1‐4	I	4/2/0/2	100	M1‐7	F1‐2	2
	2012‐1‐5	I	5/0/0/5	100	M1‐4	F1‐2	5
	2012‐1‐6	I	5/0/2/3	60	M1‐5	F1‐2	3
	2012‐2‐1^a^	II	5/0/1/4	80	M2‐3	F2‐2 F2‐3	1 3
2013	2013‐1‐1	I	5/0/4/1	20	M1‐2	F1‐1	1
	2013‐1‐2	I	5/0/0/5	100	M1‐4	F1‐2	5
	2013‐1‐3	I	4/1/3/0	0	M1‐6	/	/
	2013‐1‐4	I	4/0/0/4	100	M1‐5	F1‐2	4
	20131‐5^a^	I	8/0/3/5	62.5	M1‐1	F1‐1 F1‐2	2 3
	2013‐1‐6^a^	I	3/0/0/3	100	M1‐3	F1‐1 F1‐2	2 1
	2013‐1‐7	I	3/0/1/2	66.7	M1‐7	F1‐1	2
	2013‐2‐1^a^	II	6/0/3/3	50	M2‐5	F2‐1 F2‐2	2 1
	2013‐2‐2^a^	II	5/0/1/4	80	M2‐3	F2‐1 F2‐3	2 2
Total			79/9/18/52	74.3			

Note

Hatching success = Hatched eggs/(Total eggs − Destroyed eggs) × 100%.

a These clutches have multiple paternities; *N*_1_/*N*_2_/*N*_3_/*N*_4_: Number of total/destroyed/undeveloped/hatched eggs.

### 3.2 Paternity analysis

Nine microsatellite loci were successfully amplified in the 20 parents and 52 hatchlings. The average number of alleles per locus in all the samples was 3.89, the mean expected heterozygosity was 0.541, and the mean polymorphic information content (PIC) was 0.4071. Paternality was assigned to each of the 52 hatchlings at the 95% confidence interval (Table 3). Multiple paternity was found in 6 of the 15 viable clutches (40%); 2 clutches showed multiple paternity (2012‐1‐1 and 2012‐2‐1) in 2012, and 4 clutches exhibited multiple paternity (2013‐1‐5, 2013‐1‐6, 2013‐2‐1, and 2013‐2‐2) in 2013 (Table 3). All of the multiple paternity clutches were sired by two males. The nine remaining clutches exhibited single paternity.

**Figure 2:**
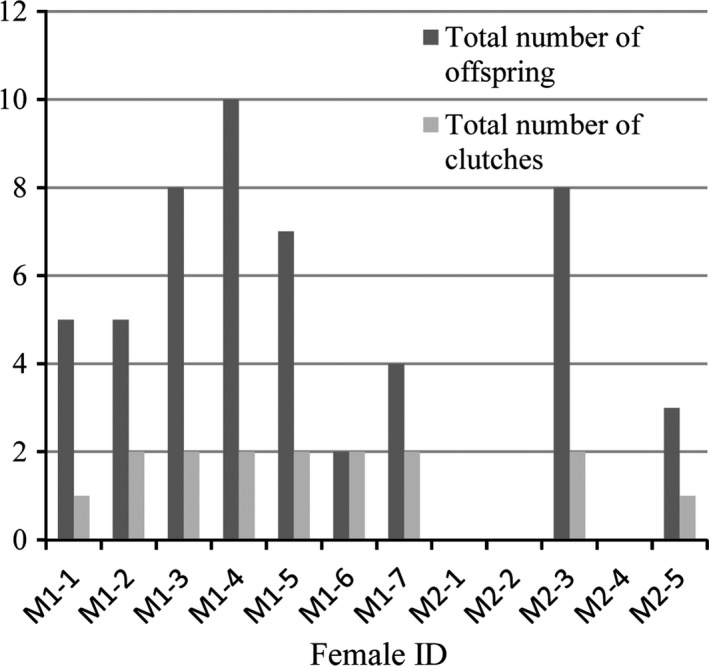
The number of clutches and offsping of 12 female *Platysternon megacephalum*

**Figure 3:**
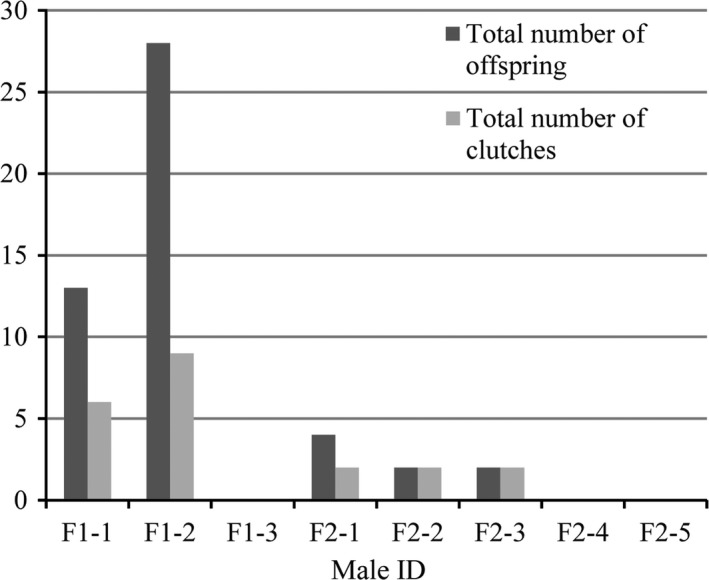
Paternal contributions from 8 males to 52 *Platysternon megacephalum* hatchlings

Nine females (7 in pond I, 2 in pond II) and five males (2 in pond I, 3 in pond II) participated in successful reproduction. For the females, all of the females in pond I laid eggs, of which one female (M1‐1) laid one clutch, and each of the other 6 females (M1‐2, M1‐3, M1‐4, M1‐5, M1‐6, M1‐7) laid two clutches. Only 2 of the 5 females in pond II laid eggs; one female (M2‐3) laid two clutches, and the other one (M2‐5) laid one clutch (Figure 2). For the males, 2 (F1‐1, F1‐2) of the 3 males in pond I contributed to 12 clutches, 3 (F2‐1, F2‐2, F2‐3) of the 5 males in pond II contributed to 3 clutches, and the remaining 3 males (F1‐3, F2‐4, F2‐5) made no contribution to any clutches (Figure 3). For the three multiple‐sired clutches in pond I, male F1‐1 and F1‐2 both had 5 offspring, respectively. For the three multiple‐sired clutches in pond II, F2‐1, F2‐2, and F2‐3 had 4, 2, and 5 offspring, respectively. There was no significant correlation between female body size and clutch size (*r* = .645, *p *> .05), no significant correlation between clutch size and multiple paternity (*r* = .096, *p *> .05), and no significant difference in hatching success between multiple‐sired clutches and single‐sired clutches (0.74 vs. 0.79; *p *> .05; Kolmogorov–Smirnov test). The PIC of each clutch in each microsatellite locus was calculated, while the mean PIC of the six clutches with multiple paternity and the nine clutches with single paternity was 0.414 and 0.402, respectively, and no statistical difference was detected (*t* = 0.945, *p *> .05).

### 3.3 Male social rank affects the number of offspring

All females in pond I and even the smallest female in pond II participated in successful reproduction, and 7 (78%) of the 9 females laid two clutches (Figure 2). There was no significant correlation between female body size and the number of offspring (*r* = .091, *p *> .05). For the males, however, there was a significant correlation between male body size and the number of offspring (*r* = .774, *p *< .05), with the larger males contributing to more clutches and thus producing more offspring. In pond I, the largest male (F1‐1) fathered 6 clutches with 13 offspring, the second largest male fathered 9 clutches with 28 offspring, and the small male had no offspring. In pond II, the three larger males fathered 6 clutches with 11 offspring and the two smaller males had no offspring (Figure 3).

**Figure 4:**
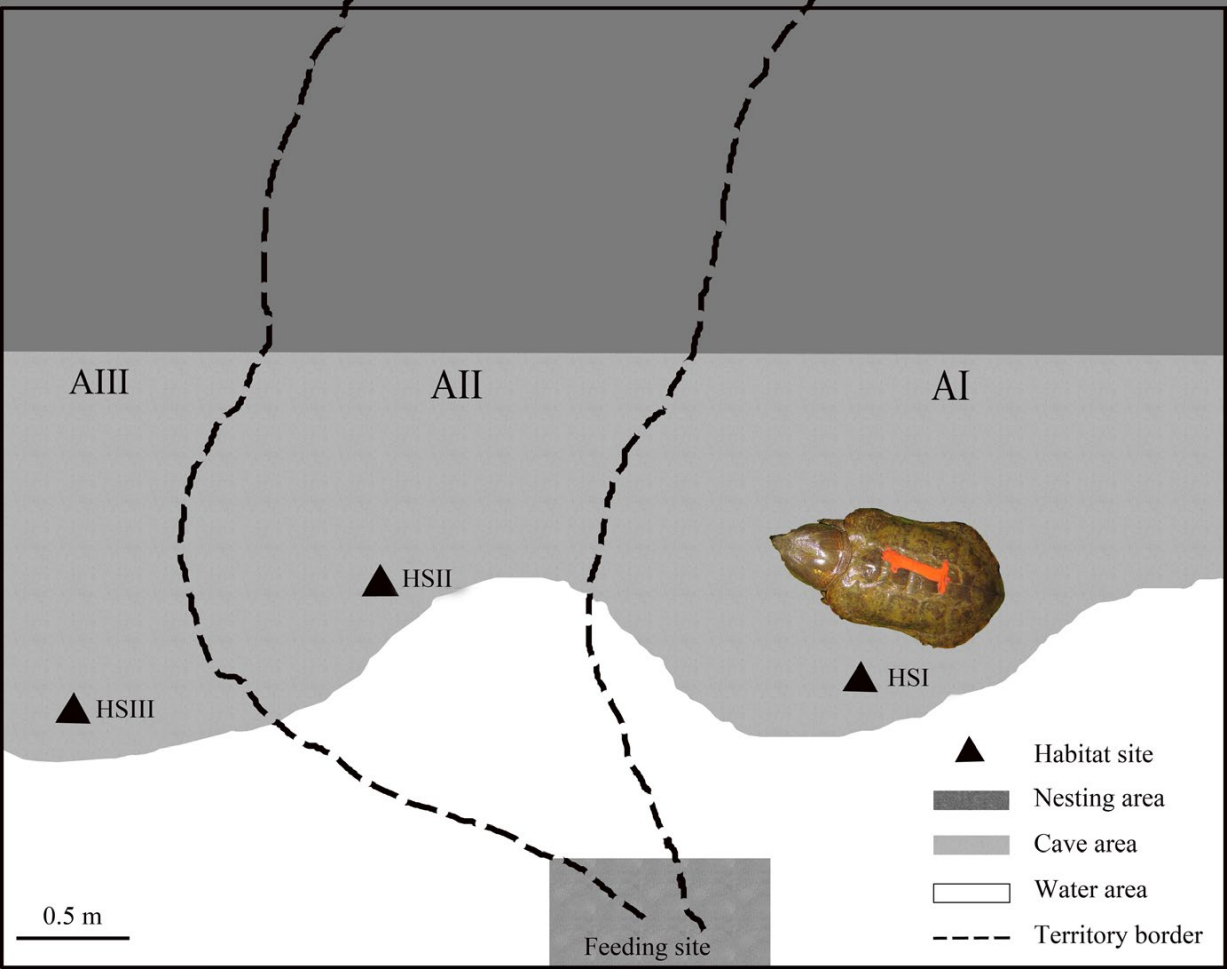
Sketch of the male turtes' territory, habitata sites, and feeding site in pond I. AI, AII, and AIII represent three regions; the bigger male (F1‐1) can move in AI, AII, and AIII; the medium‐sized male (F1‐2) can move in AII and AIII; and the smaller male (F1‐3) can move only in AIII; HS I, the habitata site of F1‐1; HS II, the habitata site of F1‐2; HS III, the habitata site of F1‐3

Behavioral observations showed that the three males in pond I had relatively fixed habitat sites (caves), but there are significant differences in their territory size. The bigger male (F1‐1), the medium‐sized male (F1‐2), and the smaller male (F1‐3) can respectively move in about 100% (AI + AII + AIII), 55% (AII + AIII), and 25% (AIII) of the area of the whole pond (Figure 4). About 45% area (AI) of the pond was only occupied by F1‐1, suggesting males with bigger body size occupy bigger territory. For the distance from habitat sites to the feeding site, F1‐1 was closest, F1‐2 was second closest, and F1‐3 was a little further away.

There were obvious eating orders among the male turtles in pond I (*χ* ^2^ = 1,011.105, *df* = 4, *p* < .01). Normally, male F1‐1, with the biggest body size, climbed to the food site first and ate. When F1‐1 left, F1‐2 began to eat, followed by F1‐3. The females had no obvious feeding order and usually took their food to secluded corners to eat. In pond II, most of the males and females ate at the same time, with no obvious eating order. However, the males and females always avoided eating together at the feeding site and they usually took their food to secluded corners to eat.

## 4 DISCUSSION

Multiple paternity has been found in some turtle species (Pearse & Avise, 2001), and our genetic data provide the first evidence of the occurrence of multiple paternity in *P. megacephalum*. Across the turtle species studied, high variability exists both in the frequency of multiple paternity and in the relative paternal contribution of males (Duran, Dunbar, Escobar, & Standish, 2015; Pearse & Avise, 2001; Roques et al., 2004). The overall incidence of multiple paternity in *P. megacephalum* was 40% (6 of 15 clutches; Table 3). Given the small sample size, this estimate for the incidence of multiple paternity may not represent the actual situation for wild populations. In this case, the incidence of multiple paternity in *P. megacephalum* is similar to that found in *Chrysemys picta* (over 30%) (Pearse, Janzen, & Avise, 2002), but lower than in *Clemmys insculpta* (50%) (Pearse & Avise, 2001), *Chelydra serpentina* (66%) (Galbraith, White, Brooks, & Boag, 1993), *Graptemys geographica* (71%) (Banger, Blouin‐Demers, Bulté, & Lougheed, 2013), and *Podocnemis expansa* (100%) (Valenzuela, 2000). The variability in the prevalence of multiple paternity in turtle species may be influenced by a variety of environmental factors, population characteristics, species behavior, and reproductive strategies. In this study, given the relatively small sample sizes, and lack of comparative data, make it difficult to determine which of these factors influence the prevalence of multiple paternity in *P. megacephalum*.

Several case studies in turtles indicated that clutches sired by multiple males contained significantly more eggs than those sired by only one male, for example, *Testudo graeca* (Roques et al., 2004) and *Chrysemys picta* (Pearse et al., 2002). These findings suggest that either a higher incidence of multiple paternity occurs in larger clutches or multiple paternity is helpful in increasing egg production. However, we did not find significant correlations between clutch size and multiple paternity in *P. megacephalum*.

Direct benefits of multiple paternity include provisioning of resources to the female and paternal care of offspring (Moller, 2000; Richardson, Burke, & Komdeur, 2002). Indirect benefits may include improving the genetic quality of the offspring (Jennions & Petrie, 2000; Yasui, 1998), increasing genetic diversity among offspring against environmental variation (Loman, Madsen, & Hakansson, 1988; Reed & Frankham, 2003), and improving genetic compatibility between mating pairs (Petrie, Doums, & Moller, 1998; Rubenstein, 2007; Tregenza & Wedell, 2000; Zeh & Zeh, 1996). Genetic incompatibilities may block egg development (Kempenaers, Congdon, Boag, Robertson, & Boag, 1999). To a given female, the variation of genetic compatibility among males may affect the hatching rate of clutches (Pearse & Avise, 2001). In turtles, without paternal care, it seems no direct benefits from the males can be given to the female in a multiple paternity system. Therefore, indirect genetic benefits may be the most likely driver of multiple matings for female turtles. In this study, there was no significant difference in hatching success between multiple‐ and single‐paternity clutches of *P. megacephalum*, and similar results were found in two other species (*Chrysemys picta*, Pearse et al., 2002; *Graptemys geographica*, Banger et al., 2013) with close evolutionary relationships (Crawford et al., 2015). The mean PIC reflects genetic diversity, and there was no significant difference in the mean PIC between multiple‐ and single‐paternity clutches of *P. megacephalum*. However, we suggest that a larger sample should be used to verify these results, especially using candidate fathers with significant genetic differences.

Male hierarchies also influence mating systems in turtles. In some turtle species, for example, *Gopherus agassizii* (Berry, 1986), *Chelydra serpentina* (Galbraith, Chandler, & Brooks, 1987), and *Clemmys insculpta* (Kaufmann, 1992), size‐based male hierarchies affect reproductive success. Males at the top of a hierarchy often occupy larger and high quality territories, and can mate with multiple females (Galbraith et al., 1993). Based on our observations in this study, size‐based male hierarchies exist in *P. megacephalum* and the males at the top of the hierarchy gained more resources (e.g., territory, nesting area, food resources) in pond I. Obviously, when females move throughout the whole pond, males with higher hierarchy have a higher mating opportunity than smaller males. However, our results indicated that the highest‐ranking males did not fertilize the most clutches, although the lowest ranking males showed no evidence of paternity in any of the clutches. This may have been caused by a combination of factors, including both male hierarchies and sexual selection.

Female choice may have an important influence on paternity in clutches of *P. megacephalum*. For a female, mating with a higher ranking male may give her the chance to share more resources. In some turtles (e.g., *Chrysemys picta*), female fecundity is related to size; larger females are more attractive to males seeking to maximize genetic fitness per mating (Pearse et al., 2002). However, in our study, there were no significant correlations between female body size and clutch size in *P. megacephalum*. Wei et al. (2016) found that a male *P. megacephalum* always avoided mating with a female larger than itself. Conversely, a female of *P. megacephalum* may prefer to mate with larger males. Therefore, female choice may also contribute to males at the top of the hierarchy having more mating opportunities. However, females also have a cryptic choice (sperm competition), among male sperm in their reproductive tracts, which may also influence the paternal contributions to clutches (Birkhead, 1998; Pearse & Avise, 2001). Sperm from the highest‐ranking males may not necessarily be the most competitive. This may explain why the highest‐ranking males in our study did not fertilize the most clutches; however, insufficient data are available to draw conclusions on this matter.

The prevalence of multiple paternity in turtles can also be influenced by a variety of environmental factors, the density of breeding individuals, the sex ratio of sexually mature individuals (Lambertucci, Carrete, Speziale, Hiraldo, & Donázar, 2013), and mate encounter rates within a reproductive season (Boomer et al., 2013; Rossi Lafferriere et al., 2016). In this case, the turtles were studied in an mimicing natural habitat (closed environment), not a real natural habitat (open environment). The density of breeding individuals of *P. megacephalum* in this study was 1 turtle per 2 m^2^, and the sex ratio of sexually mature individuals was 3 males to 7 females in pond I, and 1:1 in pond II. A higher density of adult individuals of *P. megacephalum* could lead to a higher mate encounter rate, thereby increasing the prevalence of multiple paternity. In addition, the male percentage in pond II was about 2.4 times higher than that in pond I, and the incidence of multiple paternity in pond II was 4 times higher than that in pond I. These results may imply that a higher male percentage can increase the prevalence of multiple paternity. A case study in the field in Hong Kong showed that the highest density of adult *P. megacephalum* is about 80 individuals per stream kilometer and the sex ratio is 1 male VS 0.6‐2 females (Sung et al., 2013). Obviously, the density of adult individuals of *P. megacephalum* in our study was higher than that found in the field, while the sex ratio was similar to that found in natural environments. Therefore, we speculate that the prevalence of multiple paternity of *P. megacephalum* in natural populations may be lower than the 40% found in this study.

Generally, our study under mimicing natural conditions revealed the characteristics of mating systems in *P. megacephalum* and that is helpful in further understanding the specific reproductive strategies and its possible decision mechanism. This study suggest that creating a habitat that mimics natural conditions is an effective way to achieve successful captive breeding and to investigate its mating systems of *P. megacephalum*, which provides an reference for study in other turtle species. For captive breeding of *P. megacephalum*, size‐based male hierarchies should be considered, and some low‐ranking males are not necessary in a group. Males with similar hierarchies are likely to increase the frequency of multiple paternity, potentially increasing the genetic diversity of offsprings.

## CONFLICT OF INTEREST

None declared.

## AUTHOR CONTRIBUTIONS

SG, conceived and designed research. SG, LH, and YG analyzed the data and wrote the manuscript. SG and YG collected samples and kept experimental animals. DC edited the manuscript. All authors read and approved the manuscript.

## Data Availability

All the sequence data of the nine microsatellite markers were deposited at GenBank under accessions: KC425866, KC425868, KC425870, KC425871, KC425872, KC425875, KC425876, KC425878, and KC425879. Table S1 provided the individual‐based multilocus microsatellite genotypes of all 72 individuals.
