# Supplementary material for: Unique multiple paternity in the endangered big‐headed turtle (Platysternon megacephalum) in an ex situ population in South China
Source: Ecol Evol. 2019 Aug 15;9(17):9869–77. doi: 10.1002/ece3.5528 (PMC6745651; doi:10.1002/ece3.5528)
Supplement: Supplementary file 1 [file ECE3-9-9869-s001.doc]

**Table S1 Genotypes of all 72 individuals of *Platysternon megacephalum* on the nine microsatellite loci**

| Pond# | Individual ID | Sex | Genotypes of the nine microsatellite loci | | | | | | | | |
| --- | --- | --- | --- | --- | --- | --- | --- | --- | --- | --- | --- |
|  |  |  | Pme14 | Pme42 | Pme56 | Pme59 | Pme61 | Pme112 | Pme128 | Pme156 | Pme165 |
| Pond I | F1-1 | Male | ab | ag | ab | aa | ac | ab | ab | ab | ab |
|  | F1-2 | Male | ab | ec | ab | ab | bc | aa | bc | ac | aa |
|  | F1-3 | Male | ab | ad | bb | aa | aa | aa | ab | ad | ab |
|  | M1-1 | Female | bb | ak | bf | aa | ac | ab | bc | ac | ab |
|  | M1-2 | Female | ab | ad | gh | aa | gf | aa | ab | ab | ab |
|  | M1-3 | Female | bb | de | ac | aa | ec | aa | ab | ab | bb |
|  | M1-4 | Female | ab | ad | bf | ab | ec | ab | aa | ac | aa |
|  | M1-5 | Female | bb | hi | bf | aa | ac | ab | ab | ac | aa |
|  | M1-6 | Female | bb | ag | ab | ab | eb | aa | ab | ab | aa |
|  | M1-7 | Female | bb | ad | bb | aa | ee | ab | ab | ab | bb |
| Pond II | F2-1 | Male | bb | ag | bb | aa | ec | aa | ab | ab | bb |
|  | F2-2 | Male | ab | ag | bf | aa | aa | aa | ab | ab | bb |
|  | F2-3 | Male | ab | ag | ab | aa | aa | aa | ab | ab | bb |
|  | F2-4 | Male | ab | ed | ab | aa | ee | ab | ab | ab | ab |
|  | F2-5 | Male | bb | ad | bb | aa | ab | aa | ab | ab | ab |
|  | M2-1 | Female | bb | ac | af | aa | cc | ab | ab | ac | ab |
|  | M2-2 | Female | ab | gi | bf | aa | cc | aa | ab | ab | ab |
|  | M2-3 | Female | bb | ac | bf | aa | ae | aa | bc | ac | bb |
|  | M2-4 | Female | ab | ag | ab | aa | ec | aa | ab | ab | ab |
|  | M2-5 | Female | ab | ec | aa | aa | cc | ab | ab | ab | bb |
| Year | Clutch ID | Individual ID | Pme14 | Pme42 | Pme56 | Pme59 | Pme61 | Pme112 | Pme128 | Pme156 | Pme165 |
| 2012 | 2012-1-1 | 12-1-1-1 | ab | ag | ab | ab | ae | aa | ab | bb | ab |
|  |  | 12-1-1-2 | bb | eg | aa | bb | ec | aa | bc | ab | aa |
|  | 2012-1-2 | 12-1-2-1 | ab | ad | ac | aa | ac | aa | ab | bb | ab |
|  |  | 12-1-2-2 | bb | gd | ac | aa | cc | aa | ab | bb | ab |
|  |  | 12-1-2-3 | ab | gd | ab | aa | ac | aa | ab | bb | bb |
|  |  | 12-1-2-4 | bb | gd | ac | aa | ae | aa | ab | ab | ab |
|  |  | 12-1-2-5 | ab | ad | aa | aa | ae | aa | ab | ab | ab |
|  | 2012-1-3 | 12-1-3-1 | ab | dc | ah | aa | gc | aa | bc | ab | ab |
|  |  | 12-1-3-2 | ab | ae | bh | aa | fc | aa | bc | ac | ab |
|  |  | 12-1-3-3 | bb | ae | bg | ab | gc | aa | bb | aa | aa |
|  |  | 12-1-3-4 | bb | ac | bg | ab | gc | aa | ab | aa | ab |
|  | 2012-1-4 | 12-1-4-1 | ab | ae | ab | ab | ec | aa | ab | ac | ab |
|  |  | 12-1-4-2 | ab | ae | bb | ab | ec | aa | bc | ab | ab |
|  | 2012-1-5 | 12-1-5-1 | ab | ed | bb | ab | ec | ab | ac | aa | aa |
|  |  | 12-1-5-2 | bb | dc | bb | bb | ec | ab | ab | aa | aa |
|  |  | 12-1-5-3 | bb | ed | bf | ab | ec | ab | ab | aa | aa |
|  |  | 12-1-5-4 | bb | ed | bb | ab | bc | ab | ab | ac | aa |
|  |  | 12-1-5-5 | ab | dc | bb | aa | cc | ab | ab | aa | aa |
|  | 2012-1-6 | 12-1-6-1 | ab | hc | bf | aa | cc | aa | ac | cc | aa |
|  |  | 12-1-6-2 | ab | ic | bf | ab | cc | ab | ac | aa | aa |
|  |  | 12-1-6-3 | ab | ie | af | aa | ac | aa | bc | ac | aa |
|  | 2012-2-1 | 12-2-1-1 | ab | ac | ff | aa | aa | aa | ac | ab | bb |
|  |  | 12-2-1-2 | bb | ec | bf | aa | ae | ab | bc | aa | ab |
|  |  | 12-2-1-3 | bb | dc | bb | aa | ae | ab | bc | ac | ab |
|  |  | 12-2-1-4 | ab | ec | af | aa | ae | ab | bc | ab | bb |
| 2013 | 2013-1-1 | 13-1-1-1 | bb | ag | ah | aa | fc | ab | ab | ab | bb |
|  | 2013-1-2 | 13-1-2-1 | bb | ac | bb | bb | cc | aa | ac | ac | aa |
|  |  | 13-1-2-2 | ab | ed | bb | bb | cc | aa | ab | cc | aa |
|  |  | 13-1-2-3 | ab | dc | af | ab | ec | ab | ac | ac | aa |
|  |  | 13-1-2-4 | ab | ed | ab | aa | ec | ab | ab | ac | aa |
|  |  | 13-1-2-5 | ab | ed | ab | aa | cc | ab | ab | cc | aa |
|  | 2013-1-4 | 13-1-4-1 | bb | ic | bb | ab | cc | aa | ab | ac | aa |
|  |  | 13-1-4-2 | bb | ic | bf | aa | bc | ab | bc | cc | aa |
|  |  | 13-1-4-3 | bb | hc | ab | ab | ac | aa | ac | cc | aa |
|  |  | 13-1-4-4 | ab | ei | bf | aa | ac | aa | ab | ac | aa |
|  | 2013-1-5 | 13-1-5-1 | bb | ak | af | aa | cc | ab | ab | aa | ab |
|  |  | 13-1-5-2 | bb | kc | bf | ab | bc | aa | bc | cc | ab |
|  |  | 13-1-5-3 | bb | ag | bb | aa | ac | ab | ab | ab | bb |
|  |  | 13-1-5-4 | ab | ek | bf | aa | bc | aa | bc | aa | ab |
|  |  | 13-1-5-5 | bb | kc | bf | aa | bc | ab | bc | aa | aa |
|  | 2013-1-6 | 13-1-6-1 | bb | gd | ab | aa | ae | aa | ab | ab | bb |
|  |  | 13-1-6-2 | ab | ec | ac | aa | bc | aa | ab | aa | ab |
|  |  | 13-1-6-3 | ab | eg | ab | aa | ec | aa | ab | aa | bb |
|  | 2013-1-7 | 13-1-7-1 | ab | ag | ab | aa | ec | aa | ab | ab | ab |
|  |  | 13-1-7-2 | ab | ag | bb | aa | ae | aa | ab | ab | ab |
|  | 2013-2-1 | 13-2-1-1 | bb | ae | ab | aa | ec | aa | ab | bb | bb |
|  |  | 13-2-1-2 | bb | ae | af | aa | ac | aa | ab | ab | bb |
|  |  | 13-2-1-3 | bb | ae | ab | aa | cc | aa | ab | ab | bb |
|  | 2013-2-2 | 13-2-2-1 | bb | ac | bf | aa | ac | aa | ab | ab | bb |
|  |  | 13-2-2-2 | bb | ae | bf | aa | ee | aa | ab | ab | bb |
|  |  | 13-2-2-3 | bb | ac | bb | aa | ac | aa | ab | ab | bb |
|  |  | 13-2-2-4 | bb | ae | bb | aa | ae | ab | ab | ab | bb |
